# Astragalus membranaceus (Huang Qi) for cancer-related fatigue: A protocol for systematic review and meta-analysis

**DOI:** 10.1097/MD.0000000000028633

**Published:** 2022-01-21

**Authors:** Jun Dong, Shuo Wang, Yuerong Gui, Dandan Wang, Xiumei Ma, Shuaihang Hu, Xinyan Wang, Ying Zhang, Wei Hou

**Affiliations:** Oncology Department, Guang’anmen Hospital, China Academy of Chinese Medical Sciences, Beijing, China.

**Keywords:** astragalus, cancer-related fatigue, therapeutic effect

## Abstract

**Background:**

Cancer-related fatigue (CRF) is one of the most common complications of cancer. The incidence of CRF is higher than 80%. The NCCN describes it as a distressing, persistent, subjective sense of physical, emotional, and/or cognitive tiredness or exhaustion. It exists in cancer treatment and may last for months or even years. CRF seriously impairs patients’ quality of life. However, there is still a lack of effective drug treatment. Astragalus can improve patients’ fatigue state in the clinical practice of Chinese medicine. There are some studies on the treatment of CRF with Astragalus-containing prescription. However, there is no comprehensive analysis of them. We will perform a meta-analyze on the therapeutic effect of Astragalus-containing prescription for CRF.

**Methods:**

We will search China National Knowledge Infrastructure, Chinese Scientific Journal Database, Chinese Biomedical Literature Database, Wanfang Database, EMBASE, MEDLINE, and the Cochrane Registry of Controlled Clinical Trials. The information is from the databases’ inception to December 15, 2021. According to the Cochrane Handbook for Systematic Reviews of Interventions, data extraction and processing are carried out. Review Manager 5.4 is used for meta-analysis.

**Results:**

We will take the severity of CRF as the primary outcome.

**Conclusions:**

We will conduct a meta-analysis to evaluate the efficacy of Astragalus-containing prescriptions in treating CRF.

## 1 Introduction

Cancer-related fatigue (CRF) severely impairs patients’ quality of life. The National Comprehensive Cancer Network (NCCN) defines CRF as a distressing, persistent, subjective sense of physical, emotional, and/or cognitive tiredness or exhaustion related to cancer or cancer treatment that is not proportional to recent activity and interferes with usual functioning.^[1]^ It is one of the most common complications of cancer. The incidence of CRF varies with tumors, treatments, and evaluation methods, but it is higher than 80% in most reports.^[2–4]^ The burden of CRF grows as the incidence of cancer increases year by year. CRF exists in cancer treatment and may last for months or even years.^[5]^ Its management is also an essential part of cancer treatment. Current intervention methods include exercise, psychology, and mood, anemia, and nutrition treatments.^[4,6,7]^ There is still a lack of effective treatment drugs.

Astragalus membranaceus (Huang Qi) is a kind of Chinese herbal medicine with a history of thousands of years of clinical application in China. It can invigorate the spleen and replenish qi. Astragalus is widely used in patients with Qi deficiency in traditional Chinese medicine.^[8]^ CRF is clinically manifested as fatigue, which is one of the main symptoms of Qi deficiency and can be treated with Astragalus. There are currently some clinical trials on the efficacy of Astragalus compounds in treating CRF.^[9]^ We will systematically analyze the therapeutic effect of Astragalus-containing prescription on CRF.

## 2 Methods and analysis

### 2.1 Information sources and search strategies

We will search the following databases without language limitation: China National Knowledge Infrastructure, Chinese Scientific Journal Database, Chinese Biomedical Literature Database, Wanfang Database, EMBASE, MEDLINE, the Cochrane Registry of Controlled Clinical Trials. We will search the literature from the databases’ inception to December 15, 2021. We will use a combination of subject terms and free words: “Astragalus,” “Huang Qi,” “Bei Qi,” “Huangqi,” “Astragali radix,” “Astragalus membranaceus,” “membranous milkvetch root,” “Radix Astragali,” “Fatigue,” “Asthenia,” “Fatigue Syndrome, Chronic,” “Cancer Related Fatigue,” “Quality of Life,” “Neoplasm,” “Leukemia,” “Lymphoma,” “Tumor,” “Cancer,” “Randomized controlled trial,” “Randomized,” “Placebo,” “Controlled clinical trial,” “Meta-analysis,” “Review.” Ongoing clinical trials and the reference list of published related articles will also be browsed for a supplement.

### 2.2 Inclusion criteria

1. Adults histologically diagnosed with any cancer (including all stages and treatments of cancer).
2. The intervention model is any oral Chinese medicine preparation containing astragalus. Injections are not included in the study because of the long course of CRF, and the injections are not easy to apply. Studies in combination with other therapies can be included in the analysis.
3. The control group treatment should be placebo, blank control, or other nonpharmacological therapies: sleep, exercise, music therapy, usual care, and so on.
4. Report CRF assessment as a primary or secondary research result.
5. Only randomized controlled trials (RCT) are included in the analysis.

### 2.3 Exclusion criteria

1. Screen articles that meet the inclusion criteria to ensure that the same participant does not appear in more than 1 article.
2. Full text or key data is not available.
3. Other drugs are given to CRF as research interventions.
4. Lack of extractable CRF scores.

### 2.4 Outcome measures

We take the severity of CRF as the primary outcome. In the included studies, CRF was evaluated with a scoring scale, and the treatment effect was expressed by changes in the score. We will conduct a comprehensive analysis of these changes.

### 2.5 Literature screening and data extraction

Endnote X9 will be used for literature screening. At least 2 investigators screen the researches’ title, abstract, and full text according to the criteria and import the included studies into the endnote library. Different opinions will be determined through discussion. The literature selection process follows PRISMA.

Two researchers independently extracted the included studies and entered the data into a unified Excel extraction table. Data extraction follows the Cochrane Handbook for Systematic Reviews of Interventions. Extracted information includes basic information, research design, risk assessment, population, intervention, control, CRF evaluation.^[10]^ If necessary, we will contact the author to obtain data.

### 2.6 Assess the risk of bias in the included studies

The risk of bias in the included randomized clinical trials will be independently assessed by 2 investigators (JD and DDW) by the Cochrane risk-of-bias tool. The Jadad scale will also be used to evaluate the quality of reporting, and if a trial with a score of 3 or more will be considered “high quality.”

### 2.7 Assessment of heterogeneity and reporting bias

Studies will be considered heterogeneous if the *P* value for the Chi^2^ test is <.10 or if the *I*^2^ statistic is >50% in the forest plot. We will check whether the data is entered correctly and perform subgroup analysis. A funnel plot recommended by the Cochrane Collaboration Network will be used to assess reporting bias.

### 2.8 Statistical analysis of data

Review Manager 5.4 will be used for the statistical analysis. Since the CRF evaluation results are continuous variables, if the included studies use the same scale, the mean difference will be used for meta-analysis. Otherwise, standardized mean difference analysis will be performed to merge data from different scoring scales. Ninety-five percentage confidence interval will be adopted. If the heterogeneity is low, use the fixed effects model, otherwise the random-effects model. If the data is unsuitable for meta-analysis, we will conduct a narrative review of the available evidence.

### 2.9 Analysis of subgroups or subsets

If the studies are sufficient, we will conduct a subgroup analysis. Relevant subgroups may include baseline treatment status, such as during primary treatment, after primary treatment, and baseline cancer, such as nonmetastatic, metastatic.^[11]^

### 2.10 Sensitivity analysis

We will conduct a sensitivity analysis by including only studies with a low risk of bias to see if the results are consistent under different assumptions.

### 2.11 Ethics and publishing

Ethical approval does not apply to this study because it is a secondary study, and the data is obtained from databases.

## 3 Discussion

A meta-analysis found that medication was less effective than exercise and psychological interventions, but it did not include evidence of Chinese herbal medicine.^[11]^ Given the excellent performance of Astragalus in qi deficiency syndrome, it may be effective against CRF.^[8,12]^ Astragalus is often used with other herbal medicines, such as angelica sinensis, to nourish qi and blood. When used with ginseng, atractylodes, tangerine peel, rhizoma cimicifugae, bupleurum, licorice and angelica, it forms buzhong yiqi decoction, which can strengthen the spleen and replenish qi. Astragalus is the main component for treating Qi deficiency syndrome among various prescriptions. Therefore, we chose Astragalus as an intervention and systematically analyzed the therapeutic effect of Astragalus-containing prescription for CRF.

## Author contributions

**Data curation:** Jun Dong.

**Investigation:** Jun Dong, Shuo Wang, Yuerong Gui, Dandan Wang, Xiumei Ma, Xinyan Wang.

**Methodology:** Jun Dong.

**Software:** Shuaihang Hu.

**Supervision:** Ying Zhang, Wei Hou.

**Writing – original draft:** Jun Dong.

**Writing – review & editing:** Ying Zhang, Wei Hou.
